# Patient Satisfaction With Remote Consultations in a Primary Care Setting

**DOI:** 10.7759/cureus.17814

**Published:** 2021-09-08

**Authors:** Joseph Anderson, Joanna Walsh, Martin Anderson, Rachel Burnley

**Affiliations:** 1 Faculty of Health Sciences, University of Bristol, Bristol, GBR; 2 Primary Care, Whiteladies Medical Group, Bristol, GBR; 3 Medical & Biological Sciences, University of St Andrews, St Andrews, GBR

**Keywords:** telephone consultation, patients satisfaction, remote consultations, telemedicine experience, primary care medicine

## Abstract

Introduction

In recent years, the use of remote consultations has increased considerably. Many patient encounters in general practice are now conducted by phone or computer as opposed to traditional face-to-face appointments. The aim of this study was to measure patient satisfaction with remote consultations in a primary care setting.

Aims

To assess patient satisfaction with telephone consultations in a general practice setting and explore patient’s experiences and attitudes toward remote consultations in general practice to formulate recommendations for potential telehealth improvements.

Methods

A total of 407 patients who had undergone primary care telephone consultations within the previous week were invited to provide feedback. Patient satisfaction was measured by a four-step questionnaire on patient experience, which was quantified on a Likert agreement scale, and the additional option of a comment section at the end of each questionnaire. The responses in the comment section were analysed according to the frequency of recurrent themes.

Results

The responses of 104 patients were included in the final analysis, and 44 patients used the comment section to provide additional information about their experience. Overall, the satisfaction with remote consultations was high while the rate of technical failure and the need for in-person follow-up were both low: 60 patients (58%) either agreed or strongly agreed that remote consultations are a convenient way of receiving health care and 26 patients (25%) would prefer to have remote consultations over face-to-face ones in the future while 42 patients (40%) would prefer face-to-face consultations in the future. Ninety-six (96) patients (92%) reported no technical problems affecting the consultation quality. Of all 104 face-to-face appointments, only 36 patients (35%) required in-person follow-up. Analyses of the comment section largely reflect the above findings but also highlight concerns from patients that remote consultations can generate additional anxiety, and symptoms might not be communicated effectively or even missed completely.

Discussion

The high satisfaction levels, low rates of technical failure and low need for a face-to-face follow-up show that in a primary care setting, remote consultations are an effective complement to face-to-face appointments. Nonetheless, the requirement for face-to-face contact goes beyond the need for physical examination alone, with many patients preferring face-to-face contact when discussing complex and sensitive health-related topics and symptoms.

## Introduction

The implementation of remote consultations in the form of text messages and telephone and video appointments has been ramped up significantly in recent years [1-3]. Telehealth has been shown to be a useful alternative to face-to-face appointments and has advantages for patients, health practitioners and the environment [1-6]. Today the use of remote consultations is omnipresent across medical specialities, a process greatly accelerated by the coronavirus disease 2019 (COVID-19) pandemic [2,6-8].

The Royal College of General Practitioners and other similar bodies have recently reiterated that remote consultations will form a fundamental role in future care delivery and emphasize the need for ongoing research and investment into telehealth to optimise both the provision of care and the patient experience [7,9].

The advantages of telehealth have been documented in previous papers, with much focus on investigating patients and health care professionals’ experiences and attitudes in a secondary care setting [4-5,10]. In the primary care setting, much focus has been on investigating health practitioners’ attitudes and experiences with remote consultations [6,8,11], with less emphasis on patient´s experience and satisfaction with remote consultations [12-13]. In response to the ongoing long-term transition in health care provision, this paper aims to measure patient satisfaction and attitudes concerning telehealth encounters in a general practice setting.

## Materials and methods

Patient experience was assessed using a five-step questionnaire and a further comment section at the end of the questionnaire. Each of the first four questions was rated on a five-point Likert scale. The first question screened for technical difficulties impacting the remote consultation, the second and third addressed patient convenience and satisfaction with the service and the fourth question addressed patient preference for the future appointment medium. The last question addressed whether the remote consultation was alone sufficient to address the patient’s needs or whether an additional in-person follow-up was needed to deal with any patient’s concerns.

The questions selected were based on previous papers by Fatehi et al. [14], who assessed patient satisfaction in a remote diabetic clinic, as well as Curtis et al. [4] and Graham et al. [5], who assessed patient satisfaction for remote orthopaedic consultations in a secondary care setting. The questionnaire was then reviewed and adapted by one general practitioner (GP) and three patients for clarity of content and accessibility via mobile phone. To minimise responder fatigue, the questionnaire was reduced to five questions but with an option for further comments in an open text format at the end of the questionnaire.

**Figure 1:**
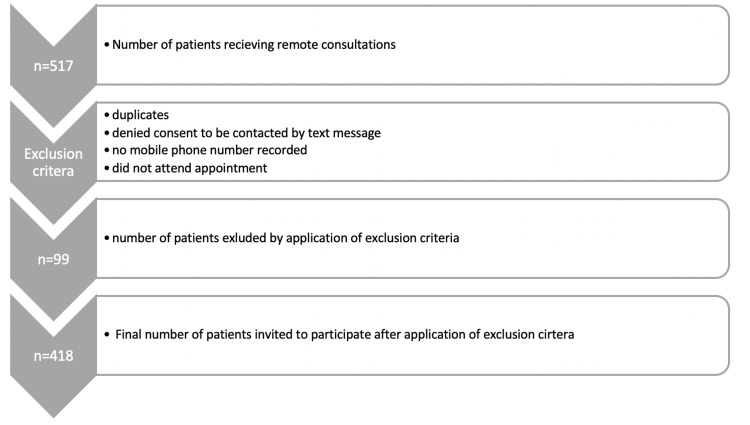
Exclusion criteria and number of patients (n) invited to participate in the study

On three separate days (May 26, 2021; July 16, 2021; and July 26, 2021), a computer-generated list was used to select all patients who had received remote consultations in the previous seven days. This generated a total cohort of 517 patients. Of this cohort, 99 patients were excluded from participating based on one of the exclusion criteria illustrated in Figure 1. A total of 418 patients were sent a text message with an invitation to participate in the study. The questionnaire was sent out a maximum of seven days after the consultation. Only responses that were submitted within seven days of receiving the invitation to participate were included in the final analysis, with the aim of reducing the chance of recall bias. All GP-led consultations performed by telephone were included, with no specific exclusion criteria for consultation content, with the aim of reflecting the diverse nature of consultation topics encountered in a primary care setting.

The invitation to participate anonymously together with a link to the questionnaire was sent out to patients via a text message from the practice computer. Results were stored centrally on the practice computer system, with no patient identifiers on the respective response forms and no record of the response visible on patients' notes. The comment section of the questionnaire was reviewed and transcribed into a single document. Recurrent themes were colour coded according to frequency.

## Results

Fifty-nine per cent (59%) of patients were female and 41% male, the age range was 17 to 97 years and the average patient age was 61.71 years (mode=69 years and median=65 years). With a response rate of 25%, a total of 104 responses were documented and included in the final analysis. Sixty-seven per cent (67%) of all responses were received on the same day they were sent out, 15% the following day, and 7% two days after being sent out. The remaining 11% were received between four and seven days after sending out the invitation to participate.

**Figure 2:**
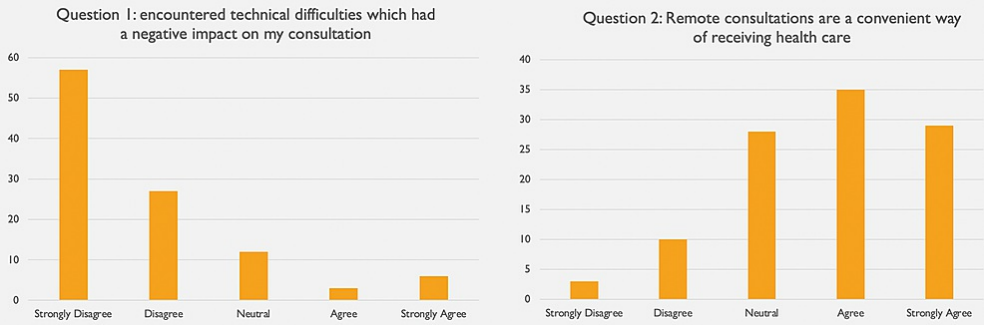
Results of question 1 assessing for technical difficulties impacting consultation quality and question 2 measuring convenience of the remote consultation service provided

**Figure 3:**
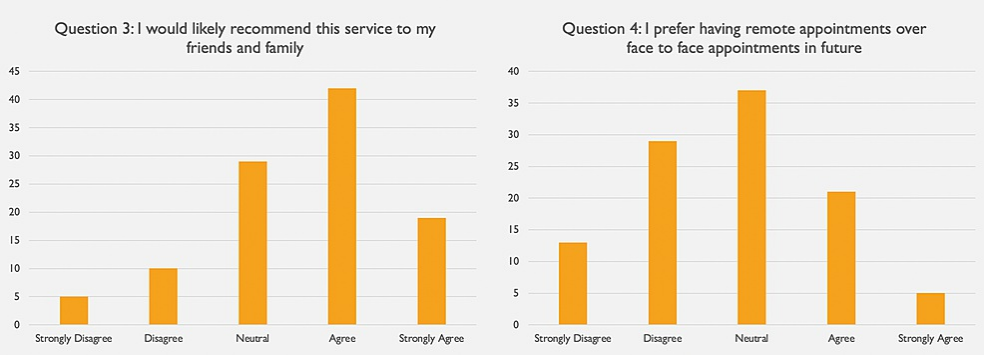
Results from question 3 assessing general patient satisfaction with remote consultations and question 4 investigating preference for future consultation type

**Figure 4:**
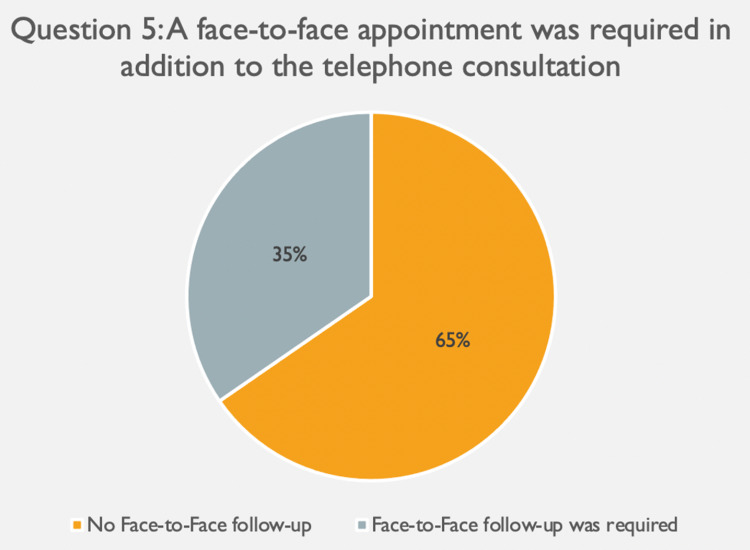
Result from question 5 of the patient questionnaire, measuring the need for face-to-face follow up following a remote consultation

The first question assessed for technical difficulties during the telephone consultation (Figure 2). Ninety-six (96) participants (92%) reported no technical difficulties affecting the consultation, with very few agreeing (3%) or strongly agreeing (6%) that the consultation was impacted by technical problems (Figure 2). Sixty-four (64) patients (62%) agreed remote consultations are a convenient way of receiving health care, with only 10 patients (10%) disagreeing and three patients (3%) strongly disagreeing (Figure 3). When asked whether they would recommend this service to family and friends, the results were comparable, with 42 patients (40%) agreeing and 19 patients (18%) strongly agreeing to this (Figure 3). Responses to the fourth question were more diverse, with 37 patients (36%) being neutral as to whether they would prefer having appointments remotely over face-to-face (Figure 3). Further, 29 patients (28%) either disagreed and 13 patients (13%) even strongly disagreed with this while 25 patients (25%) favoured having remote consultations over face-to-face ones. Of all 104 appointments, only 36 patients (35%) required in-person follow-up, with 68 patients (65%) reporting that a remote consultation was sufficient to address their needs (Figure 4).

Of all 104 responses, 44 patients made use of the comment section. Screening of comments yielded four recurrent themes (Table 1). Eight patients reported they would favour video calls over telephone calls. A further 14 responses reported a very positive experience with remote consultations, describing the experiences as either reassuring, efficient or convenient, with three patients specifically pointing out the benefits of eliminating travel time to and from the practice. On further analysis of the comment section, eight patients reported concerns regarding the possibility of incomplete information exchange during telephone consultation due to the added difficulty of communicating symptoms and concerns effectively to the health professional by telephone. Finally, nine patients expressed concerns about missing the appointment call and the inability to call back following a missed call.

**Table 1:** Illustration of recurrent themes and the number of patients commenting on the respective theme together with a selection of comments for each topic

Recurrent theme	Patients	Example comments
Preference for video over telephone calls	8	“I think it would be nice to have video calls more available when it's difficult to describe what's wrong.”
		“I think a video consultation would be much better than the telephone. Much more personal and effective, and more opportunity for the Dr to be empathetic with the patient.”
		“I think a video call would be best, adds an additional aspect to the call. Technology is with us to stay, let’s use it.”
Patient convenience and efficiency	14	“I think remote consultation is the way forward. Certainly more convenient for me.”
		“I've had three remote telephone consultations recently. They all worked well. I like the fact that I do not have to visit the surgery, this is a major convenience for me.”
Anxiety created by remote consultations resulting from an inability to communicate symptoms and worries effectively	8	“I find that too much is lost in translation when appointments are by telephone and via video. One person's idea of shape/size etc is not the same as another’s. As a patient, I would rather see someone in person. In person, it also allows for a more detailed conversation about the problem without feeling like a diagnosis means the end of the conversation and the phone call is ended before you can ask more questions.”
		“Face-to-face consultations are always best especially with older people who may be suffering the side effects of prescribed medication and lack the ability to adequately describe the symptoms which may be having long term effects unless corrected early. These are often missed with a telephone consultation which in my experience are often very brief.”
Anxiety created by remote consultations resulting from an inability to communicate symptoms and worries effectively (8 patients)	9	“Work really well. Only two slight issues are no time for calls received, and inability to call back if miss call, but I understand why that is. Wonder if some sort of “expect a call sometime between x and y” might help manage expectations? In my case, I missed 2 callbacks as wasn’t expecting/on another call.”
		“More accurate estimation of appointment time would be appreciated else it can restrict one’s activities for the whole day whereas a physical appointment used to be more convenient in this respect It should be made clearer on which number call will be made as people often have both a mobile and a landline.”

## Discussion

This study was a patient-focused evaluation of remote consultations in a UK primary care setting. Overall, our findings indicate positive patient attitudes and satisfaction with remote consultations. Fifty-eight per cent (58%) agree remote consultations are a convenient way of receiving health care, and 59% would recommend this service to a friend or family. Rates of technical failure were low, with 92% reporting no technical difficulties impacting the consultation. The effectiveness of remote consultation was additionally highlighted by the low need for face-face follow-up: Of all patients, only 35% required additional in-person appointments, suggesting that remote consultations can significantly reduce the need for face-to-face consultations. It is important to acknowledge that only immediate face-to-face follow-up appointments were picked up by this questionnaire, and all those required in subsequent weeks and months were not detected. Therefore, it could be argued that the outcome measured is an underestimate of the true number of face-to-face appointments required and thus need to be considered with caution.

The high levels of patient satisfaction are comparable to Graham et al. [5], who recorded patient satisfaction in a remote orthopaedic fracture clinic, and Imlach et al. [12], who assessed patient satisfaction with remote consultations in a primary care setting by the use of a satisfaction questionnaire and semi-structured interviews. Similar to our findings, Imlach et al. highlight convenience as a key driver for high satisfaction rates [12]. However, they also suggest that remote consultations cannot replace face-to-face appointments when a formal examination is required. Further, the study observes that remote consultations work best for routine presenting complaints and in cases where an established doctor-patient relationship is already in place [12].

The current study supports the premise previously touched on by Imlach et al. [12], that remote consultations should be regarded as an important supplement rather than a replacement of face-to-face services, with 42% of patients preferring face-to-face over remote consultations in the future. Additionally, an analysis of the comment section suggests that the justification for in-person consultations goes beyond the need for physical examination alone. Consultations in general practice often deal with multiple complex and sensitive patient concerns and expectations in a short period of time [15]. Discussing such personal information remotely can be challenging for patients, as concerns regarding effectively expressing complex topics and symptoms remotely were repeatedly highlighted by patients as downsides to remote consultations (Table 1). Additional findings specific to this study were patient concerns raised about the possibility of missing a call, not being able to call back when a call was missed and being in an inappropriate setting when receiving the call (Table 1).

The added complexity of effective and safe communication noted by patients in this study has also been reported by care providers. Murphy et al. investigated the health professional’s experiences with remote consultations in a primary care setting, illustrating that general practitioners and other specialist staff too have concerns about increased clinical risks, including the difficulty of recognising when a remote consultation alone is not sufficient in providing safe patient-centred care [2]. To reduce such concerns, they reiterate the need for clear practice policies to help health care professionals identify when telephone, video or face-to-face appointments are appropriate.

Based on the concerns reported in our study, sharing such information regarding policies and the telemedicine process with patients has the potential to reduce patient anxiety. Aiming to minimise such patient anxieties and maximising satisfaction, Table 2 implements the above learning points and provides suggestions for future remote primary care services.

**Table 2:** Learning points for the development of patient-centred telephone clinics

In a general practice setting, the choice of whether the appointment is to be held remotely or in-person is justified not only by the need for a physical examination but also by the patient’s preference and the content of the patient’s concerns.
Discussing sensitive health-related topics remotely can be challenging for patients and create additional anxiety that could, in some cases, be mitigated by a face-to-face encounter and an established doctor-patient relationship.
To manage patients’ expectations, the service provider needs to give clear advice on when and on which phone (mobile or landline) to expect the call so that the patient can prepare to communicate sensitive matters in private.

Limitations

The invitation to take part in the study was sent out via text message. To reduce recall bias, the text message was sent out between one and seven days after the telephone consultation and responses were only included if received within seven days. Ninety-nine (99) patients were excluded from the initial patient cohort, secondary to the exclusion criteria discussed above. This, together with a response rate of 25%, poses a risk of selection bias, as respondents may not be representative of the general practice population. To maximise patient responses, future studies could benefit from utilising multiple media to collect data and not limit invitations to text messages alone. Here, the invitation to participate was only sent out once via text message and no patient reminders were sent out within the seven-day period, which may have improved participation. According to Barron et al., this has the potential to compromise the accuracy of findings and introduce possible bias by underrepresenting negative patient evaluations [16].

When discussing the findings of the comment section, it is important to keep in mind that the data collected in this part of the study need to be regarded as complementary data to the patient questionnaire. It is not qualitative data per se, as it does not comply with guidelines for reporting qualitative research [17]. Nonetheless, the collection and presentation of this data is in keeping with advice by O’Cathrin et al., who provide guidance for the collection and analysis of free-text comment sections [18]. While it provides important insights into patients concerns and expectations of remote consultations, the outcomes documented here would require further validation by formal qualitative analysis in future research.

Finally, this was a single-centre study, which must be borne in mind when extrapolating to other regions and practices. Nevertheless, the challenges raised (Table 1) and recommendations in Table 2 are not region or practice specific and are therefore relevant and implementable elsewhere.

## Conclusions

Remote consultations in primary care appear to be generally well-accepted by patients and can substantially reduce the need for face-to-face appointments. However, a significant proportion of patients prefer face-to-face encounters. Beyond the absence of physical examination, barriers to effective remote consultations include difficulties in communicating complex issues and symptoms, as well as patient anxieties surrounding the practicalities of remote consultations. Educating patients on the remote consultation process could further improve the patient experience, mitigate potential barriers and maximise the benefit of remote encounters. Further exploration identifying specific patient groups most accepting and suitable for remote consultations will help primary care services target telemedicine at those most likely to benefit and streamline resource allocation.
